# Intraparenchymal breast leiomyoma and atypical leiomyoma

**DOI:** 10.1186/s12905-022-01700-6

**Published:** 2022-04-14

**Authors:** Mengping Long, Xuejiao Lina Hu, Guiyang Zhao, Yiqiang Liu, Taobo Hu

**Affiliations:** 1 Department of Pathology, Peking University Cancer Hospital, Beijing, China; 2 Department of Pathology, Alaska Native Medical Center, Anchorage, AK USA; 3 Department of Oncology, Beijing Changping Hospital, Beijing, China; 4 Department of Breast Surgery, Peking University People’s Hospital, No. 11 Xizhimen South Street, Xicheng District, Beijing, 100044 China

**Keywords:** Breast leiomyoma, Atypical leiomyoma, Case report

## Abstract

**Background:**

Breast leiomyoma is a rare benign mesenchymal tumor, accounting for less than 1% of all breast neoplasms. Cases of breast atypical leiomyoma is even more rarely reported and its diagnostic criteria together with its clinical courses is not cleared defined.

**Case presentation:**

We described two patients with breast leiomyomas. One has unilateral benign breast leiomyoma, the other one has bilateral breast leiomyomas. For the bilateral case, the left-side tumor was diagnosed as benign leiomyoma while the right-side tumor was diagnosed as atypical leiomyoma. The morphological features that lead to the diagnosis of atypical leiomyoma are its invasive growth pattern, mild nuclear atypia, and mitotic figures up to 3mitoses/10HPF.

**Conclusions:**

Atypical breast leiomyoma appears to behave like benign leiomyoma without recurrence in our study with nine-year follow-up. Due to the limited experience, cases presented as atypical intraparenchymal breast leiomyoma should be closely followed.

## Background

Breast leiomyoma is a rare breast parenchymal tumor, accounting for less than 1% of all breast neoplasms [1]. It usually occurs in middle-aged women with an average age of 47.6 years [2]. Breast leiomyoma share the same histology and immunophenotype with leiomyoma of other sites. A reliable diagnosis is better achieved on excisional specimens with characteristic morphology and immunohistochemical (IHC) staining pattern that are the same with leiomyoma from other organ origins. It is well established that there are atypical smooth muscle tumors of the uterus [3, 4]. However, due to its rarity in the breast, no definitive diagnostic criteria have been proposed for atypical breast leiomyoma [5, 6]. Here, we present two cases of breast leiomyoma and for one of them a diagnosis of atypical leiomyoma should be considered based on its infiltrating growth pattern, mild nuclear atypia and increased mitotic figures.

## Case presentation

### Case 1

**Fig. 1:**
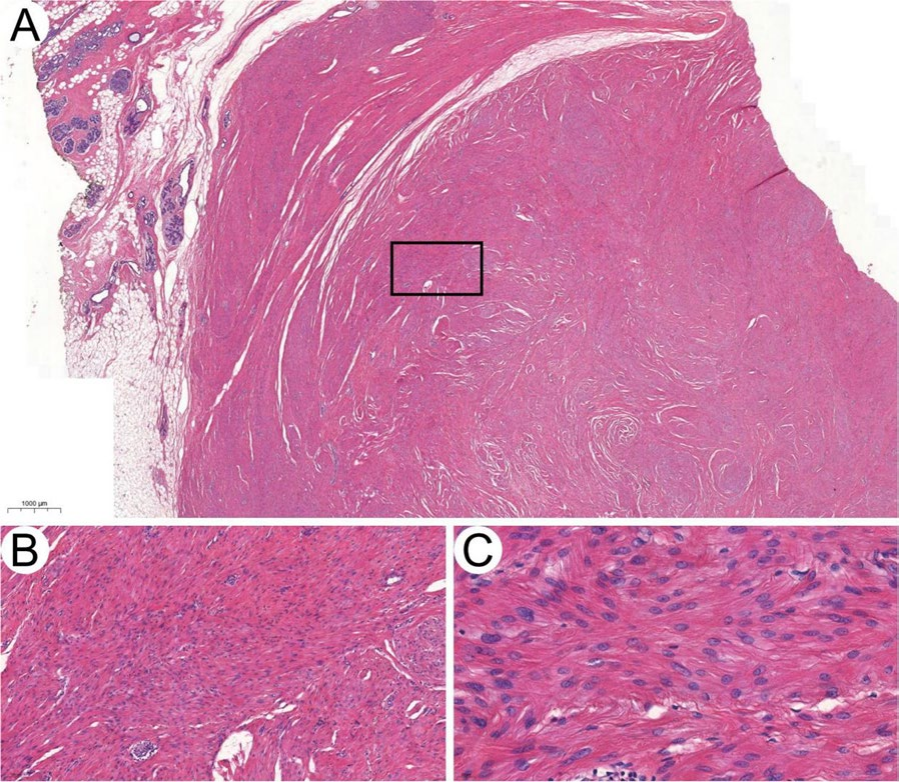
Benign intraparenchymal breast leiomyoma. **A**–**C** Histologic image of leiomyoma in case 1 (H&E × 10, × 100 and × 400)

A 46-year-old woman presented with a 3 cm left breast lump detected ten days ago in March 2019. Lumpectomy was performed with all margins clean. Pathology examination revealed a well-circumscribed dense tan mass in breast parenchyma. Histologic examination showed monotonous spindle cell proliferation without nuclear atypia, necrosis or mitotic figures found (Fig. 1A–C). These cells are uniformly positive for smooth muscle actin (SMA), desmin, and caldesmon in IHC staining, and the Ki-67 index was smaller than 1%. The diagnosis of intraparenchymal breast leiomyoma was made. There is no recurrence after 2.5 years of follow-up.

### Case 2

A 43-year-old woman presented with relapsed bilateral breast lumps for six months in September 2012. Ultrasound revealed a 1 cm mass in the right breast and a 3 cm one in the left. She had a history of bilateral breast masses in 2010, which were excised by lumpectomies and pathology diagnosis was spindle cell tumor without further classification.

**Fig. 2:**
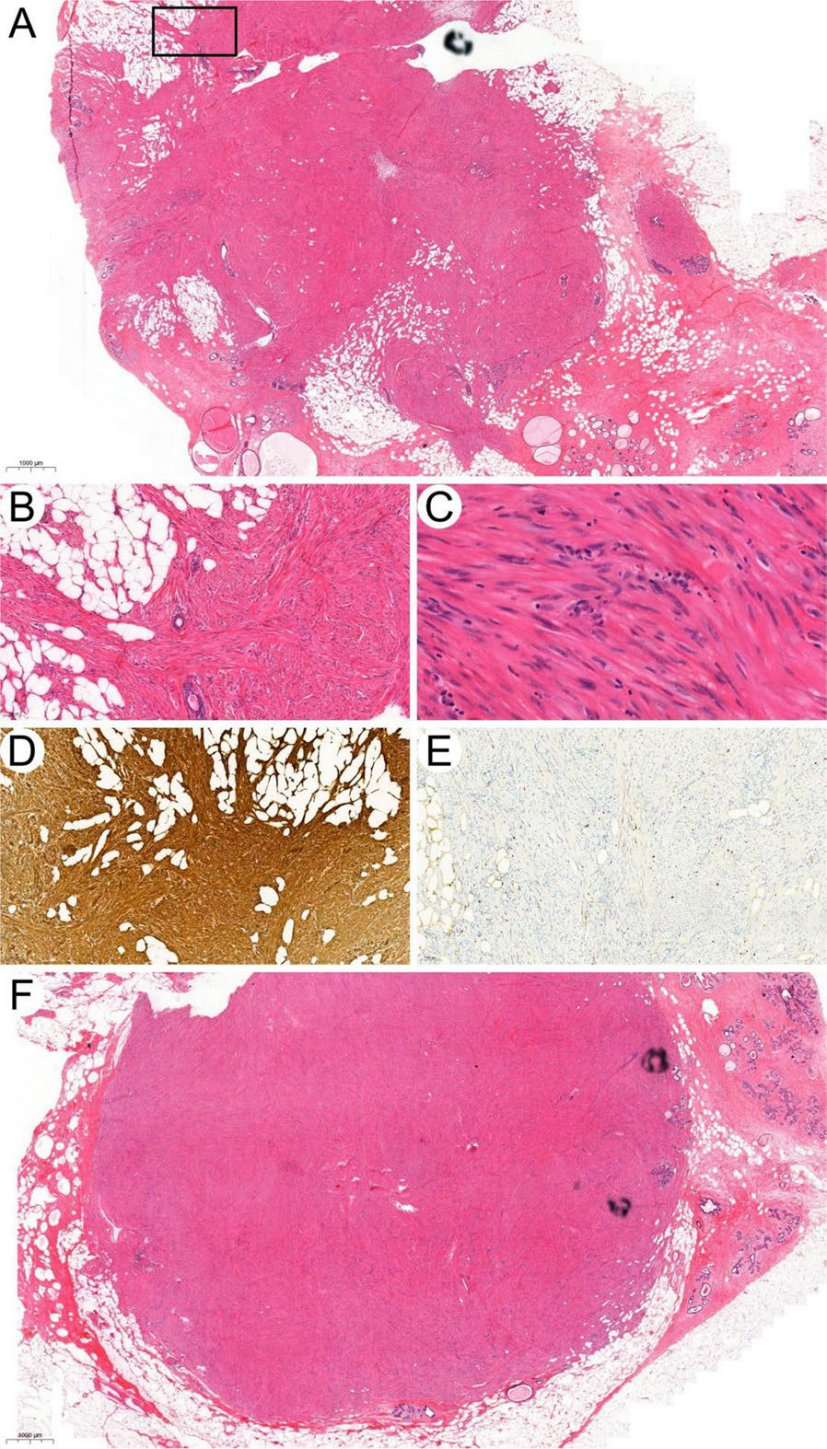
Bilateral leiomyoma of breast. **A** Leiomyoma of left breast. Noted the invasive growth pattern of this tumor including its infiltrative margin and the packed normal mammary glands in it. The infiltrative margin indicated by the black square is magnified in B (H&E, × 10). **B** Noted the infiltration of this tumor into adipose tissue (H&E, × 200). **C** Nuclei of tumor cells in high magnificent view showed typical spindle morphology with blunt end which is characteristics for leiomyoma (H&E, × 400). **D**, **E** Immunohistochemical stain of SMA and Ki-67 in leiomyoma of left breast. **F** Leiomyoma of right breast (H&E, × 10)

Bilateral lumpectomies were performed this time with all margins clean. No further treatment was applied. The histopathology of the left breast mass displayed an infiltrating growth pattern with mild nuclear atypia and mitotic figures up to 3/10 HPF (Fig. 2A–C), which have exceeded the diagnostic criteria for typical benign leiomyoma but fall short of the threshold for leiomyosarcoma. Therefore, the diagnosis of atypical leiomyoma was made. The simultaneous right breast tumor has similar histopathological features with the left side but without apparent infiltration or increased mitosis (Fig. 2F). A diagnosis of intraparenchymal breast leiomyoma was made for the right-side mass. Both tumors share the same IHC staining pattern with positive SMA and desmin. The Ki-67 index is about 5% in atypical leiomyoma (Fig. 2D, E). The patient has no recurrence until the last follow-up in July 2021.

## Discussion and conclusion

Leiomyoma of the breast is rare and atypical breast leiomyoma is even rarer. To the best of our knowledge, no case of atypical breast leiomyoma has been reported in the English-based literature. Thus, the biological behavior, recurrence rate or diagnostic criteria of atypical breast leiomyoma is unclear. In our reported case of atypical leiomyoma, the patient has no recurrence after lumpectomy by nine years of follow-up, indicating a potential benign behavior of atypical breast leiomyoma. The most common treatment for breast leiomyoma is resection with clean margins [7, 8]. In our experience with atypical leiomyoma, patients achieved favorable prognosis with wide excision only. On account of the limited experience, cases presented as atypical intraparenchymal breast leiomyoma should still be closely followed after surgery.

Due to its rarity, atypical breast leiomyoma has no diagnostic consensus. In uterine, atypical leiomyoma is termed as Uterine Smooth Muscle Tumor of Uncertain Malignant Potential (STUMP) which is also rare. The diagnosis criteria of STUMPs include focal/multifocal or diffuse nuclear atypia and 6–9 mitoses/10HPF; more than 15 mitoses/10HPF without cytological atypia or necrosis; tumor with diffuse nuclear atypia and uncertain mitoses which is often due to brisk karyorrhexis [9–13].

The diagnostic criteria of breast leiomyosarcoma are similar to other parts of the body based on the degree of infiltration, nuclear atypia, and mitotic activity [14–16]. The morphology of atypical leiomyoma falls between leiomyoma and leiomyosarcoma. In multiple or recurrent breast leiomyoma, hereditary leiomyomatosis and renal cell cancer (HLRCC) due to germline mutation of fumarate hydratase should be excluded [17], although our patient had no such family history. The differential diagnosis of breast leiomyoma includes fibroblastic and myofibroblastic tumor, spindle cell lipoma, nodular fasciitis, and nerve sheath tumor [18]. Usually, positive IHC stains for SMA and desmin would confirm the smooth muscle origin. The cell origin of breast parenchymal leiomyoma is not entirely clear; several hypotheses including smooth muscle metaplasia of myoepithelial cell, from smooth muscle cells of the blood vessel or derived from differentiation of multipotent mesenchymal cells has been proposed [19].

## Data Availability

The datasets used during the current study available from the corresponding author on reasonable request.

## Abbreviations

HLRCC: Hereditary leiomyomatosis and renal cell cancer
HPF: High power field
IHC: Immunohistochemistry
SMA: Smooth muscle actin
STUMP: Uterine smooth muscle tumor of uncertain malignant potential
